# Editorial: Zoonoses-a rising threat to healthcare system

**DOI:** 10.3389/fmicb.2023.1232183

**Published:** 2023-06-13

**Authors:** Wenn-Chyau Lee, Yang Cheng, Varakorn Kosaisavee, Laurent Renia

**Affiliations:** ^1^Department of Parasitology, Faculty of Medicine, Universiti Malaya, Kuala Lumpur, Malaysia; ^2^A^*^STAR Infectious Diseases Labs (A^*^STAR ID Labs), Agency for Science, Technology and Research (A^*^STAR), Singapore, Singapore; ^3^Institute of Biomedical Sciences, Academia Sinica, Taipei, Taiwan; ^4^Department of Parasitology and Entomology, Faculty of Public Health, Mahidol University, Bangkok, Thailand; ^5^Lee Kong Chian School of Medicine, Nanyang Technological University, Singapore, Singapore; ^6^School of Biological Sciences, Nanyang Technological University, Singapore, Singapore

**Keywords:** zoonoses, infectious diseases, parasite, virus, bacteria

Zoonoses are infections caused by pathogens that are transmitted from animals to humans. They contribute to significant healthcare burden in many parts of the world. The incidence of spillover infections from animals to humans may increase and spread to wider geographical areas in future, due to the changes of climate, ecology, population structure, and socioeconomic activities (Ellwanger and Chies, 2021; Lee et al., 2022). Additionally, immigration and traveling further complicate the transmission biology of zoonoses (Mavroidi, 2008), imposing challenges to the management and control of such outbreaks. Notably, many zoonotic pathogens cause asymptomatic infections to their natural hosts but produce severe pathology in humans (Owen et al., 2004; Evangelista and Coburn, 2010; Hu et al., 2022). As healthcare workers may not be familiar with the diagnosis and pathogenesis of different zoonoses in humans, delayed clinical interventions are relatively common, compromising prognosis. Importantly, research attention dedicated to many zoonotic outbreaks has been shown to wane over time. Thus, a Research Topic of articles covering different aspects of several zoonoses and infections with animal reservoirs were brought together, to offer a convenient reference platform for scientists and healthcare workers.

Monkeypox was undeniably one of the most concerning zoonoses in 2022. Panda and Mukherjee provided their opinions regarding the transmission dynamics of monkeypox in humans, as well as the treatment and management of this infection. Bragazzi et al. compiled a mini review on factors that lead to the underestimation of sexually transmitted diseases, with a special focus on monkeypox. In addition, Ullah et al. put together a comprehensive review article on the epidemiology of monkeypox and its potential threat to public health sector. In contrast to monkeypox that received relatively high public attention, leptospirosis is a low key, yet highly fatal bacterial zoonosis. To better understand the pathobiology of *Leptospira* infection, Pětrošová et al. investigated the structural diversity of *Leptospira* lipid A, the hydrophobic component of endotoxin that is responsible for much of the endotoxin toxicity. Adding to this, van der Westhuizen et al. studied the prevalence of occupational exposure of farmworkers to zoonotic pathogens such as *Brucella* sp., hantaviruses, and *Leptospira* sp. in South Africa.

This Research Topic also received a number of articles related to several zoonotic parasites, some of which are neglected tropical diseases. *Fasciola gigantica* is a large liver fluke of ruminants that readily infects humans, causing fascioliasis. Zheng et al. deciphered the proteins that constituted *F. gigantica* excretory and secretory products (FgESP) derived from the sera of infected buffalos at different time points of infection. Mano et al. reported the correlation between amphotericin B resistance and the increased fitness of *Leishmania martiniquensis*, an autochthonous vector-borne zoonosis in Thailand. Phang et al. (a) investigated *Plasmodium knowlesi*, a potentially fatal vector-borne zoonosis that is prevalent in Southeast Asia. The team predicted the transmission risk of *P. knowlesi* by using machine learning-based ecological niche modeling approaches. A corrigendum for this work was also published by Phang et al. (b) in this Research Topic. Akoolo et al. reviewed the influence of protozoan coinfections on the efficacy of vaccines against the bacterial and viral pathogens. Several coinfection models with relevance to human epidemiological situation were highlighted, such as the coinfection of *Plasmodium* and non-typhoidal *Salmonella* (an important group of zoonotic bacteria), Rotavirus and *Cryptosporidium* coinfection, as well as *Babesia* spp. and *Borrelia burgdorferi* coinfection (both are vector-borne zoonoses). In addition, Wong et al. presented a review on vector management in the control and elimination of vector-borne zoonoses and vector-borne infections with animal reservoirs.

Zoonosis transmission is a broad topic with various knowledge gaps remained to be filled. Obviously, the articles assembled in this Research Topic do not fully reflect the complete picture of this Research Topic. Nevertheless, this article Research Topic contributed new insights and knowledge to this field, which may inspire new studies to improve the understanding on the transmission biology of zoonoses.

## Author contributions

W-CL drafted the editorial. YC, VK, and LR contributed to the editing. All authors provided the final approval of the version to be published.
